# Supplementary material for: Element-centric clustering comparison unifies overlaps and hierarchy
Source: Sci Rep. 2019 Jun 12;9:8574. doi: 10.1038/s41598-019-44892-y (PMC6561975; doi:10.1038/s41598-019-44892-y)
Supplement: Supplementary file 1 — Supplemental Information [file 41598_2019_44892_MOESM1_ESM.pdf]

Supplemental Information

Element-centric clustering comparison unifies overlaps and hierarchy

Alexander J. Gates[1], Ian B. Wood[2,3], William P. Hetrick[4], and Yong-Yeol Ahn[2,3,5]
[1]Department of Physics, Northeastern University. Boston, MA
[2]Department of Informatics, Indiana University. Bloomington, IN
[3]Center for Complex Networks and Systems Research, Indiana University. Bloomington, IN
[4]Department of Psychological and Brain Sciences, Indiana University. Bloomington, IN
[5]Program in Cognitive Science, Indiana University. Bloomington, IN

Contents

S1 Clusterings 2

S2 Existing measures of clustering similarity 2
S2.1 Rand Index . . . 2
S2.2 Adjusted Rand index (ARI) . . . 3
S2.3 Omega index . . . 3
S2.4 Jaccard index . . . 4
S2.5 F measure . . . 4
S2.6 Fowlkes-Mallows index . . . 4
S2.7 Percentage Matching . . . 4
S2.8 Normalized mutual information (NMI) . . . 4
S2.9 Overlapping NMI (ONMI) . . . 6
S2.10 Variation of Information VI . . . 6
S2.11 Information Theoretic Intuition . . . 7

S3 Datasets 7
S3.1 Point clusters . . . 7
S3.2 Handwriting digits . . . 8
S3.3 Brain networks . . . 8

References 9

S1 Clusterings

Throughout this work, we focus on the grouping of elements (i.e. data points or vertices) into clusters (the groups). The set of clusters is called a *clustering*. Specifically, given a set of N distinct elements $V = \{v_1, \ldots, v_N\}$, a clustering is a set $\mathscr{C} = \{C_1, \ldots, C_{K_{\mathscr{C}}}\}$ of $K_{\mathscr{C}}$ non-empty subsets of V such that every element v_i in V is in at least one cluster C_β: $\forall v_i \in V \ \exists C_\beta$ s.t. $v_i \in C_\beta$.

We consider three classes of clusterings. A *partition*, or disjoint clustering, is a clustering in which all elements are members of one, and only one, cluster. An *overlapping* clustering allows elements to be members of multiple clusters. *Hierarchical* clusterings capture the nested organization of clusters at different scales and are accompanied by a directed acyclic graph (or dendrogram) showing the hierarchical relationships between clusters.

S2 Existing measures of clustering similarity

Here, we focus on ten of the most prominent measures from the clustering literature: the Rand index, the adjusted Rand index, the Omega index, the Jaccard index, the F measure, the Fowlkes Mallows index, percentage matching (PM), normalized mutual information (NMI), overlapping normalized mutual information (ONMI), variation of information (VI). All of these measures are implemented in the CluSim python package[1].

S2.1 Rand Index

The Rand index[2] counts the number of element pairs which are either members of the same cluster, or members of different clusters in both clusterings. The most common formulation of the Rand index focuses on the following four sets of the $\binom{N}{2}$ element pairs: N_{11} the number of element pairs which are grouped in the same cluster in both clusterings, N_{10} the number of element pairs which are grouped in the same cluster by $\mathscr{A}$ but in different clusters by $\mathscr{B}$, N_{01} the number of element pairs which are grouped in the same cluster by $\mathscr{B}$ but in different clusters by $\mathscr{A}$, and N_{00} the number of element pairs which are grouped in different clusters by both $\mathscr{A}$ and $\mathscr{B}$. Intuitively, N_{11} and N_{00} are indicators of the agreement between the two clusterings, while N_{10} and N_{01} reflect the disagreement between the clusterings.

The aforementioned pair counts are identified from the contingency table $\mathscr{T}$ between two clusterings, shown in Table S1, by the following set of equations:

$$
\begin{aligned}
N_{11} &= \sum_{k,m=1}^{K_{\mathscr{A}},K_{\mathscr{B}}} \binom{n_{km}}{2} = \frac{1}{2}\left(\sum_{k,m=1}^{K_{\mathscr{A}},K_{\mathscr{B}}} n_{km}^2 - N\right) \qquad \text{(S1)} \\
N_{10} &= \sum_{k=1}^{K_{\mathscr{A}}} \binom{a_k}{2} - N_{11} = \frac{1}{2}\left(\sum_{k=1}^{K_{\mathscr{A}}} a_k^2 - \sum_{k,m=1}^{K_{\mathscr{A}},K_{\mathscr{B}}} n_{km}^2\right) \\
N_{01} &= \sum_{m=1}^{K_{\mathscr{B}}} \binom{b_m}{2} - N_{11} = \frac{1}{2}\left(\sum_{m=1}^{K_{\mathscr{B}}} b_m^2 - \sum_{k,m=1}^{K_{\mathscr{A}},K_{\mathscr{B}}} n_{km}^2\right) \\
N_{00} &= \binom{N}{2} - N_{11} - N_{10} - N_{01}.
\end{aligned}
$$

$\mathscr{A}/\mathscr{B}$	B_1	B_2	$\ldots$	$B_{K_{\mathscr{B}}}$	Sums
A_1	n_{11}	n_{12}	$\ldots$	$n_{1K_{\mathscr{B}}}$	a_1
A_2	n_{21}	n_{22}	$\ldots$	$n_{2K_{\mathscr{B}}}$	a_2
$\vdots$	$\vdots$	$\vdots$	$\ddots$	$\vdots$	$\vdots$
$A_{K_{\mathscr{A}}}$	$n_{K_{\mathscr{A}}1}$	$n_{K_{\mathscr{A}}2}$	$\ldots$	$n_{K_{\mathscr{A}}K_{\mathscr{B}}}$	$a_{K_{\mathscr{A}}}$
Sums	b_1	b_2	$\ldots$	$b_{K_{\mathscr{B}}}$	$\sum_{ij} n_{ij} = N$

Table S1. The contingency table $\mathscr{T}$ for two clusterings $\mathscr{A} = \{A_1, \ldots, A_{K_{\mathscr{A}}}\}$ and $\mathscr{B} = \{B_1, \ldots, B_{K_{\mathscr{B}}}\}$ of N elements, where $n_{ij} = |A_i \cap B_j|$ are the number of elements in both cluster $A_i \in \mathscr{A}$ and cluster $B_j \in \mathscr{B}$.

The Rand index between clusterings $\mathscr{A}$ and $\mathscr{B}$, $RI(\mathscr{A},\mathscr{B})$ is then given by the function:

$$RI(\boldsymbol{A},\boldsymbol{B}) = \frac{N_{11}+N_{00}}{\binom{N}{2}}. \tag{S2}$$

It lies between 0 and 1, where 1 indicates the clusterings are identical and 0 occurs for clusters which do not share a single pair of elements (this only happens when one clustering is the full set of elements and the other clustering groups each element into its own singleton cluster). As the number of elements being clustered becomes large, the measure becomes dominated by the number of pairs which were classified into different clusters (N_{00}), resulting in decreased sensitivity to co-occurring element pairs[3].

S2.2 Adjusted Rand index (ARI)

A popular extension of the Rand index, called the adjusted Rand index (ARI), considers the average of the measure in the context of the permutation model for random clusterings[4–6]. In the permutation model the number and size of clusters within a clustering are fixed; all random clusterings are generated by shuffling the elements between the fixed clusters. The expectation of the Rand index with respect to the permutation model follows from the fact that the entries in Table S1 follow a generalized hypergeometric distribution. Taking $Q^{\mathscr{A}} = \sum_{k=1}^{K_{\mathscr{A}}} \binom{a_k}{2}$ and $Q^{\mathscr{B}} = \sum_{m=1}^{K_{\mathscr{B}}} \binom{b_m}{2}$, the expectation $\mathbb{E}_{perm}[RI(\mathscr{A},\mathscr{B})]$ of the Rand index with respect to the permutation model for the cluster size sequences of clusterings $\mathscr{A}$ and $\mathscr{B}$ is given by:

$$\mathbb{E}_{perm}[RI(\mathscr{A},\mathscr{B})] = \frac{Q^{\mathscr{A}}Q^{\mathscr{B}} - \binom{N}{2}\left(Q^{\mathscr{A}}+Q^{\mathscr{B}}\right) + \binom{N}{2}^2}{\binom{N}{2}^2} \tag{S3}$$

(see Fowlkes and Mallows[3], Hubert and Arabie[4], or Albatineh and Niewiadomska-Bugaj[5] for the full derivation). Finally, the ARI between clusterings $\mathscr{A}$ and $\mathscr{B}$ is given by:

$$\text{ARI}(\mathscr{A},\mathscr{B}) = \frac{R(\mathscr{A},\mathscr{B}) - \mathbb{E}_{perm}[RI(\mathscr{A},\mathscr{B})]}{1-\mathbb{E}_{perm}[RI(\mathscr{A},\mathscr{B})]} \tag{S4}$$

S2.3 Omega index

The Omega index extends the adjusted Rand index to compare overlapping clusterings[7]. To formulate the extension, notice that in the presence of overlaps, element pairs can repeatedly occur within the same cluster. We consider $t_j(\mathscr{A})$ the set of node pairs which co-occur exactly j times in clustering $\mathscr{A}$. The unadjusted Omega index between two overlapping clusterings is then:

$$\omega_u(\mathscr{A},\mathscr{B}) = \frac{1}{\binom{N}{2}} \sum_j |t(\mathscr{A}) \cap t(\mathscr{B})|, \tag{S5}$$

while the expectation of this measure with respect to the permutation model on the number of element pair overlaps is:

$$\mathbb{E}_{perm}[\omega_u(\mathscr{A},\mathscr{B})] = \frac{1}{\binom{N}{2}^2} \sum_j |t(\mathscr{A})| \cdot |t(\mathscr{B})| \tag{S6}$$

Finally, the Omega index between two overlapping partitions is given by:

$$\Omega(\mathscr{A},\mathscr{B}) = \frac{\omega_u(\mathscr{A},\mathscr{B}) - \mathbb{E}_{perm}[\omega_u(\mathscr{A},\mathscr{B})]}{1-\mathbb{E}_{perm}[\omega_u(\mathscr{A},\mathscr{B})]}. \tag{S7}$$

Note that for partitions the Omega index is equivalent to the adjusted Rand index.

S2.4 Jaccard index

Another popular clustering similarity measure which utilizes pair-wise co-occurrence between the elements is the Jaccard index or Jaccard similarity coefficient[8]. Originally proposed to compare regional floras[9], the Jaccard index is a similarity measure for finite sets. It is defined as the number of element pairs which are grouped in the same cluster in both clusterings divided by the number of element pairs which are grouped in the cluster in at least one of the clusterings. Thus, it ignores the number of element pairs that are grouped into different clusters by both clusterings. One minus the Jaccard index is a metric on the collection of finite sets[10]. Using the above notation from the contingency table Table S1, the Jaccard index between clusterings $\mathscr{A}$ and $\mathscr{B}$ takes the form:

$$\mathrm{J}(\mathscr{A},\mathscr{B}) = \frac{N_{11}}{N_{11}+N_{10}+N_{01}} \tag{S8}$$

S2.5 F measure

The F measure has a long history of use in clustering validation, natural language processing, information retrieval, and machine learning. It is based off of two asymmetric measures (sometimes called Dice's asymmetric coefficients), that count the proportion of element pairs which are correctly co-assigned to the same cluster in one of the clusterings using the other clustering as a baseline. When one of these clusterings is considered to be a ground-truth clustering, these asymmetric measures are known as *precision* and *recall*. The F measure is the harmonic mean of the precision and recall. Specifically, the F measure between clusterings $\mathscr{A}$ and $\mathscr{B}$ is given by:

$$F(\mathscr{A},\mathscr{B}) = \frac{2N_{11}}{2N_{11}+N_{10}+N_{01}} \tag{S9}$$

The F measure F and Jaccard index J are related by $J = F/(2-F)$.

S2.6 Fowlkes-Mallows index

The Fowlkes-Mallows index was first introduced to facilitate the comparison of hierarchical dendrograms[3]. The idea is to cut the dendrogram at each merger and compare the induced flat clusterings. Like the previous five measures, the Fowlkes-Mallows index is based on counting the pair-wise co-occurrence between the elements in the two clusterings:

$$\mathrm{FM}(\mathscr{A},\mathscr{B}) = \frac{N_{11}}{\sqrt{(N_{11}+N_{10})(N_{11}+N_{01})}}. \tag{S10}$$

Applying this index to each cut k of two dendrograms produces a curve of comparisons between two clusterings each with k clusters.

S2.7 Percentage Matching

The Percentage Matching is based on the idea that each cluster should be compared to only one other cluster, its "best match"[11]. Specifically, let $K_{\min} = \min(K_{\mathscr{A}}, K_{\mathscr{B}})$, then the percentage matching index is defined using the contingency table:

$$\mathrm{PM}(\mathscr{A},\mathscr{B}) = 1 - \frac{1}{N}\sum_{k=1}^{K_{\min}} \max_{\pi} n_{k,\pi}. \tag{S11}$$

where the notation $\max_{\pi}$ denotes finding the cluster π with the largest overlap to cluster k. The percentage matching is equal to one minus the Purity Index, another common measure of the distance between clusterings.

S2.8 Normalized mutual information (NMI)

Another family of approaches for finding the similarity of two cluster coverings is based on the amount of information in each covering and the amount of information one covering contains about the other. These quantities can also be calculated from the contingency Table S1. The entropy H of a clustering $\mathscr{A}$ is given by

$$H(\mathscr{A}) = -\sum_{k=1}^{K_{\mathscr{A}}} \frac{a_k}{N}\log\frac{a_k}{N} \tag{S12}$$

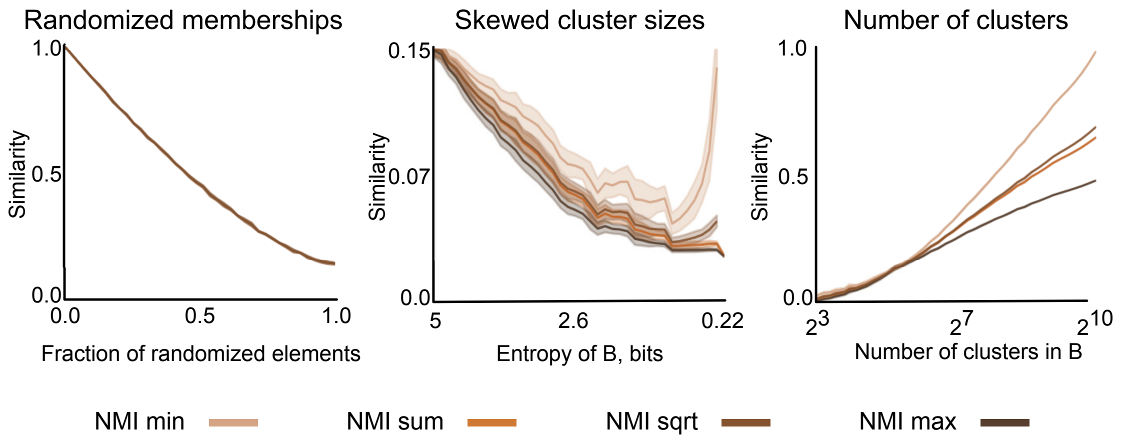

Figure S1. NMI's bias towards the number of clusters is independent of normalization term. The three scenarios from the main text, for different normalization terms of NMI: the minimum of cluster entropies (min), the average of the cluster entropies (sum), the geometric mean of the cluster entropies (sqrt), and the maximum of the cluster entropies (max). See Section S2.8 for the measure details.

and, using the entries n_{km} from the contingency table S1, the joint entropy between two clusterings $\mathscr{A}$ and $\mathscr{B}$ is

$$H(\mathscr{A},\mathscr{B}) = -\sum_{k,m=1}^{K_{\mathscr{A}},K_{\mathscr{B}}} \frac{n_{km}}{N} \log \frac{n_{km}}{N} \tag{S13}$$

Thus, the mutual information between two clusterings is given by:

$$\begin{aligned} MI(\mathscr{A},\mathscr{B}) &= H(\mathscr{A}) + H(\mathscr{B}) - H(\mathscr{A},\mathscr{B}) \\ &= \sum_{k,m=1}^{K_{\mathscr{A}},K_{\mathscr{B}}} \frac{n_{km}}{N} \log \frac{n_{km}N}{a_k b_m}. \end{aligned} \tag{S14}$$

The mutual information can be interpreted as an inverse measure of independence between the clusterings, or a measure of the amount of information each clustering has about the other. As it can vary in the range $[0, \min\{H(\mathscr{A}), H(\mathscr{B})\}]$, to facilitate comparisons, it is desirable to normalize it to the range $[0,1]$. There are at least six proposals in the literature for this upper bound, each with different advantages and drawbacks;

$$\begin{aligned} &\min\{H(\mathscr{A}),H(\mathscr{B})\} \leq \sqrt{H(\mathscr{A})H(\mathscr{B})} \leq \frac{H(\mathscr{A})+H(\mathscr{B})}{2} \\ &\leq \max\{H(\mathscr{A}),H(\mathscr{B})\} \leq \max\{\log K_{\mathscr{A}}, \log K_{\mathscr{B}}\} \leq \log N. \end{aligned} \tag{S15}$$

The resulting measures are all known as normalized mutual information (NMI). Here, we always use the average of the two clustering entropies $\frac{1}{2}(H(\mathscr{A})+H(\mathscr{B}))$. Although some results have been shown to depend on the normalization term used for NMI, Figure S1demonstrates that NMI behaves un-intuitively regardless of the normalization term.

Due to the known bias of NMI towards clusterings with more clusters, several modifications have been proposed. The NMI can be adjusted for chance according to an appropriate random model[6,12], but this induces the problem of selecting a random model for the clusterings, and does not remove the issue of selecting a normalization term. Alternatively, the NMI can be re-scaled by an exponential factor reflecting the difference in number of clusters between the two clusterings, but this scaling factor forces the researcher to prioritize one clustering as the 'ground-truth' and breaks the symmetry of the original measure[13].

S2.9 Overlapping NMI (ONMI)

The NMI has been modified to account for clusterings with overlapping clusters[14]. Consider a clustering $\mathscr{A}$ with $K_{\mathscr{A}}$ possibly overlapping clusters $A_1,\ldots,A_{K_{\mathscr{A}}}$. For each cluster A_k, we can consider a binary random variable X_k which represents the probability that a randomly selected node is a member of that cluster with distribution

$$P(X_k=1)=\frac{a_k}{N},\quad P(X_k=0)=1-\frac{a_k}{N} \tag{S16}$$

The same holds for a second clustering $\mathscr{B}$ with $K_{\mathscr{B}}$ possibly overlapping clusters $B_1,\ldots,B_{K_{\mathscr{B}}}$ and random variables Y_m. We can then define the joint probability distribution $P(X_k,Y_m)$:

$$\begin{aligned}
P(X_k=1,Y_m=1)&=\frac{n_{km}}{N}\\
P(X_k=0,Y_m=0)&=1-\frac{n_{km}}{N}\\
P(X_k=1,Y_m=0)&=\frac{a_k-n_{km}}{N}\\
P(X_k=0,Y_m=1)&=\frac{b_m-n_{km}}{N}
\end{aligned} \tag{S17}$$

Given a particular cluster $A_k\in\mathscr{A}$, the amount of information it has about another cluster $B_m\in\mathscr{B}$ is found by the conditional entropy

$$H(X_k|Y_m)=H(X_k,Y_m)-H(Y_m). \tag{S18}$$

In the case of overlapping clusters, there are many possible candidates for the best match between two clusters. The best match is the one with the minimal conditional entropy. Thus, the conditional entropy of X_k with respect to all of the clusters in $\mathscr{B}$ is

$$H(X_k|\boldsymbol{Y})=\min_{m\in\{1,\ldots,M\}}H(X_k|Y_m). \tag{S19}$$

However, in minimizing the entropy it may be the case that the optimal B_m^* is the complement of A_k, thus we have to add the following constraint to insure the above minimization identities the best matching cluster:

$$h[P(1,1)]+h[P(0,0)]>h[P(0,1)]+h[P(1,0)]. \tag{S20}$$

This entropy is normalized by the entropy of X_k and averaged over all X_k to give the normalized conditional entropy of $\boldsymbol{X}$ with respect to $\boldsymbol{Y}$

$$H(\boldsymbol{X}|\boldsymbol{Y})_{\text{norm}}=\frac{1}{K}\sum_{k=1}^{K}\frac{H(X_k|\boldsymbol{Y})}{H(X_k)}. \tag{S21}$$

Finally, the overlapping normalized mutual information ONMI is given by

$$ONMI(\mathscr{A},\mathscr{B})=1-\frac{1}{2}[H(\boldsymbol{X}|\boldsymbol{Y})_{\text{norm}}+H(\boldsymbol{Y}|\boldsymbol{X})_{\text{norm}}]. \tag{S22}$$

It is interesting to note that when $\mathscr{A}$ and $\mathscr{B}$ are partitions, the $NMI(\mathscr{A},\mathscr{B})$ and $ONMI(\mathscr{A},\mathscr{B})$ do not necessarily agree. Although there have been several attempts to reformulate ONMI so that it agrees with NMI, the above formulation is pervasive in the literature[15–17].

S2.10 Variation of Information VI

Another popular clustering comparison measure based on information theory is the Variation of Information (VI). Unlike the similarity measures discussed above, the VI is a metric on the lattice of partitions[18]. Thus, it is a measure of distance between clusterings instead of a similarity between the clusterings; it attains its minimum at 0 when the clusterings are

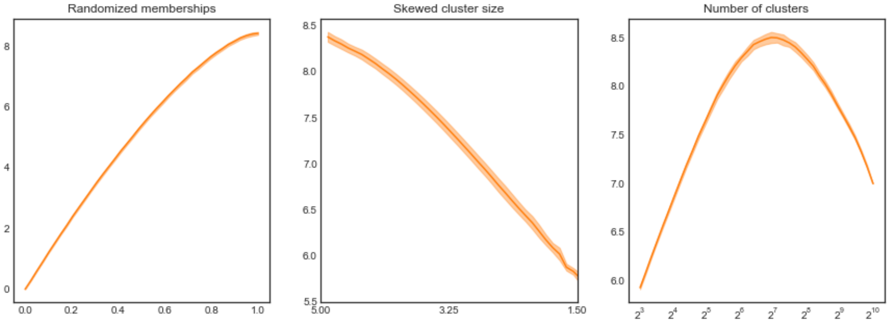

Figure S2. VI unintuitive behavior as the cluster sizes become more skewed and as the number of clusters is increased. Note that because the VI is a distance measure, the intuitive behavior is opposite that presented for similarity measures.

identical, and attains positive values for clusterings which differ. Using the entropy and mutual information between clusterings defined in Section S2.8, the VI is given by:

$$\begin{aligned} VI(\mathscr{A},\mathscr{B}) &= H(\mathscr{A}) + H(\mathscr{B}) - 2MI(\mathscr{A},\mathscr{B}) \\ &= 2H(\mathscr{A},\mathscr{B}) - H(\mathscr{A}) - H(\mathscr{B}). \end{aligned} \tag{S23}$$

Since the VI is a distance measure, the intuitive behavior is opposite that presented for the similarity measures discussed in this paper, and presented in the main text, Figure 2. None-the-less, we can demonstrate that the VI suffers from unintuitive behavior in two scenarios: the skewed cluster sizes and the number of clusters (Figure S2).

S2.11 Information Theoretic Intuition

A second intuition that could be used to evaluate clustering similarity measures is based on concepts drawn from information theory. Under this intuition, the appropriate question to ask is: "Given a random element, how much uncertainty remains about its membership in Clustering $\mathscr{B}$ if I know its membership in Clustering $\mathscr{A}$?" The clusters are now considered as an alphabet and the contingency table is considered as a discrete probability distribution over this alphabet. For example, the variation of information considers the difference in conditional entropies reflecting the amount of information we loose about the original cluster assignment, and the amount of information we have to gain to recover the new cluster assignment when going from one clustering to the other[18]. The resulting intuition suggests that two clusterings are similar if one doesn't loose much information (presence of equally sized clusters) or one doesn't have to gain much information (presence of very small clusters). Consequently, in Figure S2, we notice that the VI decreases (more similar) as the cluster entropy decreases, and displays a parabolic shape (more similar, to less similar, to more similar) as the number of clusters approaches the number of elements.

Our main objection to the information theoretic intuition is that it tends to suggest measures cannot differentiate the influence of alphabet size (here, number of clusters) from the distribution of alphabet usage (here, sizes of the clusters). Furthermore, the primary justification for these measures is typically stated with respect to the alignment to the lattice of partitions[19], yet, it is not immediately clear if the lattice of partitions is the appropriate space to compare clustering similarity measures since many applications do not align to the lattice (i.e. evaluation of k-means clustering fixes the number of clusters).

S3 Datasets

S3.1 Point clusters

$5,000$ points were random formed into clusters in an algorithm akin to the process for constructing benchmark graphs[20]. Cluster sizes were randomly drawn from a powerlaw distribution with a minimum cluster size of 10, a maximum cluster size of 1000, and an exponent of 1.0. The center of those clusters was uniformly selected from points in a 40×40 box. The standard deviation (or spread) of each cluster was also drawn from a powerlaw distribution with a minimum

of 0.2, a maximum of 2.0, and an exponent of 1.0. Next, the type of each cluster was uniformly selected from four options. The first option is the 2-D Gaussian blob with mean given by the cluster center and standard deviation given by the cluster standard deviation. The second option is the 2-D Anisotropic blob with a mean given by the cluster center, standard deviation given by the cluster standard deviation, and transformation given by the rotational matrix:

$$\begin{bmatrix} a\cos(\theta) & -a\sin(\theta) \\ b\sin(\theta) & b\cos(\theta) \end{bmatrix}, \tag{S24}$$

where a, b randomly drawn from the unit interval and θ was randomly drawn from the range $[0, \pi]$. The third option is the circle centered at the cluster center with radius given by the cluster standard deviation; the points were uniformly spread along the circle and Gaussian noise with mean 0 and standard deviation 0.2 was added to all points. The forth option is the spiral with points uniformly spread in the range $[0, 10]$, converted to circular coordinates by $(x, y) \to (\sigma\sqrt{x}\cos(x), \sigma\sqrt{y}\cos(y))$, where σ is the cluster standard deviation, randomly rotated by the rotation matrix of equation (S24) with $a = b = 1$ and θ randomly drawn from the range $[0, \pi]$, and Gaussian noise with mean 0 and standard deviation 0.2 was added to all points.

The sci-kit learn[21] implementation of K-means clustering was initialized with $K = 19$ clusters and random initial centroids. The identification method was then run from 100 random centroid initializations. Clustering agreement was calculated by comparing all 100 uncovered clusterings with the ground-truth clustering using the element-wise similarity vector was found for each comparison and then averaged over the uncovered clusterings. Clustering frustration was calculated from all pair-wise comparisons between the 100 uncovered clusterings using the element-wise similarity vector was found for each comparison and then averaged over each comparison.

S3.2 Handwriting digits

The digits data set, originally assembled by Alimoglu and Alpaydin[22], is bundled with the[21] source code. It consists of 1797 images of 8×8 gray level pixels for handwritten digits distributed across 10 clusters corresponding to the true digit. To provide a visualization, the data was projected to 2-d using the t-Distributed Stochastic Neighbor Embedding (t-SNE) dimensionality reduction method[23] initialized from the pca decomposition.

The sci-kit learn[21] implementation of K-means clustering was initialized with $K = 10$ clusters and random initial centroids. The identification method was then run from 100 random centroid initializations. Clustering agreement was calculated by comparing all 100 uncovered clusterings with the ground-truth clustering using the element-wise similarity vector was found for each comparison and then averaged over the uncovered clusterings. Clustering frustration was calculated from all pair-wise comparisons between the 100 uncovered clusterings using the element-wise similarity vector was found for each comparison and then averaged over each comparison.

S3.3 Brain networks

The dataset used here was originally analyzed in Cheng et al.[24]; please refer to that work for specific details of the data acquisition and pre-processing, here we only provide a brief overview.

Data was acquired from 19 individuals diagnosed with schizophrenia (6 female, mean age 33.1 ± 10.9 years) and 29 healthy controls (15 female, mean age 28.1 ± 8.4 years). Diagnosis of schizophrenia was based on the Structured Clinical Interview for the DSM-IV Axis I Disorders (SCID-I)[25] and medical chart review. All subjects were scanned on a Siemens TIM Trio 3 T MRI scanner using a 32-channel head coil. The high anatomical scan had a resolution of 1 mm^3. A total of 200 volumes of resting state fMRI data were acquired with EPI sequences for 8 min and 20s. During the resting state fMRI scan, the subjects were at rest with eyes closed and instructed not to think of anything in particular. All functional data were motion corrected in FSL.

In conjunction with the anatomical image, the functional images were parcellated using a parcellation scheme proposed by Shen et al.[26]. This parcellation divides the cerebral cortex into 278 regions of interest (ROIs), and was derived from resting state functional data of the healthy subjects by maximizing functional homogeneity within each ROI. After regressing out head motion, the time signal was band-pass filtered between $0.01 - 0.10$ Hz and the time courses were extracted from the 278 brain ROIs as the average over voxels.

The functional network was computed from the wavelet coherence between all pair-wise combinations of ROIs, giving rise to a square symmetric matrix (278×278). The resulting functional connectivity matrix has only positive edges. In order to identify a backbone network structure, the multiscale network backbone[27] was extracted using an

alpha of $\alpha = 0.2$. Technically, the multiscale backbone is a directed network, however, since our original graph was undirected, we convert the mutliscale backbone back into an undirected network. The network was not corrected to insure a single connected component.

Overlapping and hierarchically structured clusterings were derived using Order Statistics Local Optimization Method (OSLOM) network community detection[28] with the following parameters: weighted, undirected edges, $p = 0.1$, 100 runs for the detection at the bottom of the hierarchy and 1000 runs for the detection at the top of the hierarchy. All singlet communities were kept in the clusterings. Due to the variability in clustering structure between runs of the algorithm, 10 clusterings were extracted for each patient.

The subject similarity matrix was then constructed as follows. The similarity of each diagonal entry is 1.0. Each off-diagonal entry in the (48×48) subject similarity matrix is the average element-centric similarity similarity of all comparisons $10 \times 10 = 100$ between the 10 OSLOM communities uncovered for each subject. For all comparisons, we set $\alpha = 0.9$ and $r = 8.0$. Our choice of the scaling parameter, $r = 8.0$, was grounded in the explorations of synthetic binary hierarchies of equivalent height. The dis-similarity matrix is one minus the similarity matrix. Six additional matrices were found by using the community structure found by slicing each OSLOM community dendrogram and retaining only the bottom or top communities and performing all pair-wise comparisons with either our element-centric similarity measure, ONMI or the Omega index. Note that we use only these three measure of similarity because the communities contain many overlapping structures.

Given a dis-similarity matrix, a distance weighted k-Nearest Neighbors (kNN) classifier was trained using nested and stratified 10-fold validation[29]. Specifically, the data was randomly split into 10 groups such that the proportions of each class were kept relatively equal in each group. Each group in turn was then used as the testing set, while the other 9 groups formed the training set. For each training set, we first find the best k for the kNN classifier using a grid search for k between 1 and 15 and another stratified 10-fold validation. The classifier was then retrained on the entire training set for the specified k. Finally, the accuracy of the trained classifier was found on the testing set. In the paper, we report the average accuracy identified in 100 random initializations of the nested 10-fold validation technique[30,31].

References

1. Gates, A. J. & Ahn, Y.-Y. Clusim: a python package for calculating clustering similarity. *J. Open Source Softw.* **4**, 1264 (2019).
2. Rand, W. M. Objective Criteria for the Evaluation of Clustering Methods. *J. Am. Stat. Assoc.* **66**, 846 (1971).
3. Fowlkes, E. B. & Mallows, C. L. A method for comparing two hierarchical clusterings. *J. Am. Stat. Assoc.* **78**, 553–569 (1983).
4. Hubert, L. & Arabie, P. Comparing partitions. *J. Classif.* **2**, 193–218 (1985).
5. Albatineh, A. N., Niewiadomska-Bugaj, M. & Mihalko, D. On similarity indices and correction for chance agreement. *J. Classif.* **23**, 301–313 (2006).
6. Gates, A. J. & Ahn, Y.-Y. The impact of random models on clustering similarity. *J. Mach. Learn. Res.* **18**, 1–28 (2017).
7. Collins, L. M. & Dent, C. W. Omega: A general formulation of the rand index of cluster recovery suitable for non-disjoint solutions. *Multivar. Behav. Res.* **23**, 231–242 (1988).
8. Ben-Hur, A., Elisseff, A. & Guyon, I. A stability based method for discovering structure in clustered data. In *Pacific Symposium on Biocomputing*, 6–17 (2002).
9. Jaccard, P. The distribution of the flora in the alpine zone. *New Phytol.* **11**, 37–50 (1912).
10. Marczewski, E. & Steinhaus, H. On a certain distance of sets and the corresponding distance of functions. *Colloquium Math.* **6**, 319–327 (1958).
11. Meila, M. & Heckerman, D. An experimental comparison of model-based clustering methods. *Mach. Learn.* **42**, 9–29 (2001).
12. Vinh, N. X., Epps, J. & Bailey, J. Information theoretic measures for clusterings comparison: is a correction for chance necessary? In *Proceedings of the 26th Annual International Conference on Machine Learning*, 1073–1080 (ACM, 2009).

13. Amelio, A. & Pizzuti, C. Is normalized mutual information a fair measure for comparing community detection methods? In *Proceedings of the 2015 IEEE/ACM International Conference on Advances in Social Networks Analysis and Mining 2015*, 1584–1585 (ACM, 2015).

14. Lancichinetti, A., Fortunato, S. & Kertesz, J. Detecting the overlapping and hierarchical community structure in complex networks. *New J. Phys.* **11**, 033015 (2009).

15. Esquivel, A. V. & Rosvall, M. Comparing network covers using mutual information. *arXiv preprint arXiv:1202.0425* (2012).

16. Xie, J., Kelley, S. & Szymanski, B. K. Overlapping community detection in networks: The state-of-the-art and comparative study. *ACM Comput. Surv. (csur)* **45**, 43 (2013).

17. Hric, D., Darst, R. K. & Fortunato, S. Community detection in networks: Structural communities versus ground truth. *Phys. Rev. E* **90**, 062805 (2014).

18. Meilă, M. Comparing clusterings by the variation of information. In *Learning Theory and Kernel Machines*, 173–187 (Springer, 2003).

19. Meila, M. Comparing clusterings: an axiomatic view. In *Proceedings of the 22nd International Conference on Machine Learning*, 577–584 (ACM, New York, NY, USA, 2005).

20. Lancichinetti, A. & Fortunato, S. Community detection algorithms: a comparative analysis. *Phys. Rev. E* **80**, 056117 (2009).

21. Pedregosa, F. *et al.* Scikit-learn: Machine learning in Python. *J. Mach. Learn. Res.* **12**, 2825–2830 (2011).

22. Alimoglu, F. & Alpaydin, E. Methods of combining multiple classifiers based on different representations for pen-based handwritten digit recognition. In *Proceedings of the Fifth Turkish Artificial Intelligence and Artificial Neural Networks Symposium* (1996).

23. Van der Maaten, L. & Hinton, G. Visualizing data using t-sne. *J. Mach. Learn. Res.* **9**, 85 (2008).

24. Cheng, H. *et al.* Nodal centrality of functional network in the differentiation of schizophrenia. *Schizophr. Res.* **168**, 345–352 (2015).

25. First, M. B. *Structured clinical interview for DSM-IV-TR Axis I disorders: Patient edition* (Biometrics Research Department, Columbia University, 2005).

26. Shen, X., Tokoglu, F., Papademetris, X. & Constable, R. T. Groupwise whole-brain parcellation from resting-state fmri data for network node identification. *Neuroimage* **82**, 403–415 (2013).

27. Serrano, M. A., Boguna, M. & Vespignani, A. Extracting the multiscale backbone of complex weighted networks. *PNAS* **106**, 6483–6488 (2009).

28. Lancichinetti, A., Radicchi, F., Ramasco, J. J. & Fortunato, S. Finding Statistically Significant Communities in Networks. *PLoS ONE* **6**, e18961 (2011).

29. Hastie, T., Tibshirani, R. & Friedman, J. The elements of statistical learning. *NY Springer* (2001).

30. Stone, M. Cross-validatory choice and assessment of statistical predictions. *J. Royal Stat. Soc. Ser. B (Methodological)* 111–147 (1974).

31. Rao, R. B., Fung, G. & Rosales, R. On the dangers of cross-validation. an experimental evaluation. In *SDM*, 588–596 (SIAM, 2008).
